# Atherosclerosis treatment with nanoagent: potential targets, stimulus signals and drug delivery mechanisms

**DOI:** 10.3389/fbioe.2023.1205751

**Published:** 2023-06-19

**Authors:** Ting Luo, Zhen Zhang, Junbo Xu, Hanxiong Liu, Lin Cai, Gang Huang, Chunbin Wang, Yingzhong Chen, Long Xia, Xunshi Ding, Jin Wang, Xin Li

**Affiliations:** ^1^ Department of Cardiology, The Third People’s Hospital of Chengdu Affiliated to Southwest Jiaotong University, Key Laboratory of Advanced Technologies of Materials Ministry of Education, Southwest Jiaotong University, Chengdu, Sichuan, China; ^2^ School of Materials Science and Engineering, Southwest Jiaotong University, Chengdu, China; ^3^ Institute of Biomedical Engineering, College of Medicine, Southwest Jiaotong University, Chengdu, Sichuan, China

**Keywords:** atherosclerosis, nanoagent, targets, stimulus signals, drug delivery

## Abstract

Cardiovascular disease (CVDs) is the first killer of human health, and it caused up at least 31% of global deaths. Atherosclerosis is one of the main reasons caused CVDs. Oral drug therapy with statins and other lipid-regulating drugs is the conventional treatment strategies for atherosclerosis. However, conventional therapeutic strategies are constrained by low drug utilization and non-target organ injury problems. Micro-nano materials, including particles, liposomes, micelles and bubbles, have been developed as the revolutionized tools for CVDs detection and drug delivery, specifically atherosclerotic targeting treatment. Furthermore, the micro-nano materials also could be designed to intelligently and responsive targeting drug delivering, and then become a promising tool to achieve atherosclerosis precision treatment. This work reviewed the advances in atherosclerosis nanotherapy, including the materials carriers, target sites, responsive model and treatment results. These nanoagents precisely delivery the therapeutic agents to the target atherosclerosis sites, and intelligent and precise release of drugs, which could minimize the potential adverse effects and be more effective in atherosclerosis lesion.

## 1 Introduction

Atherosclerosis (AS) is a chronic disease characterizedby the deposition of lipid in vascular tissues. It is the common pathological basis for ischemic cardiovascular disease (CVDs), including stroke, coronary artery disease, cerebrovascular disease, and peripheral arterial disease, which pose a serious threat to human health (Alfarisi et al., 2020; Mariano et al., 2020; Maedeh et al., 2021; Wong et al., 2022). It is estimated that approximately 17.7 million people die from CVDs each year, accounting for 31% of global deaths (Libby et al., 2016; Ma et al., 2018). The China Cardiovascular Health and Disease Report 2020 states (Report on Cardiovascular Health and Diseases in China, 2021, 2022) that the number of cardiovascular patients in China is now around 330 million, and the prevalence of CVDs is still rising. CVDs continue to pose a significant public health challenge in both China and globally.

**FIGURE 1:**
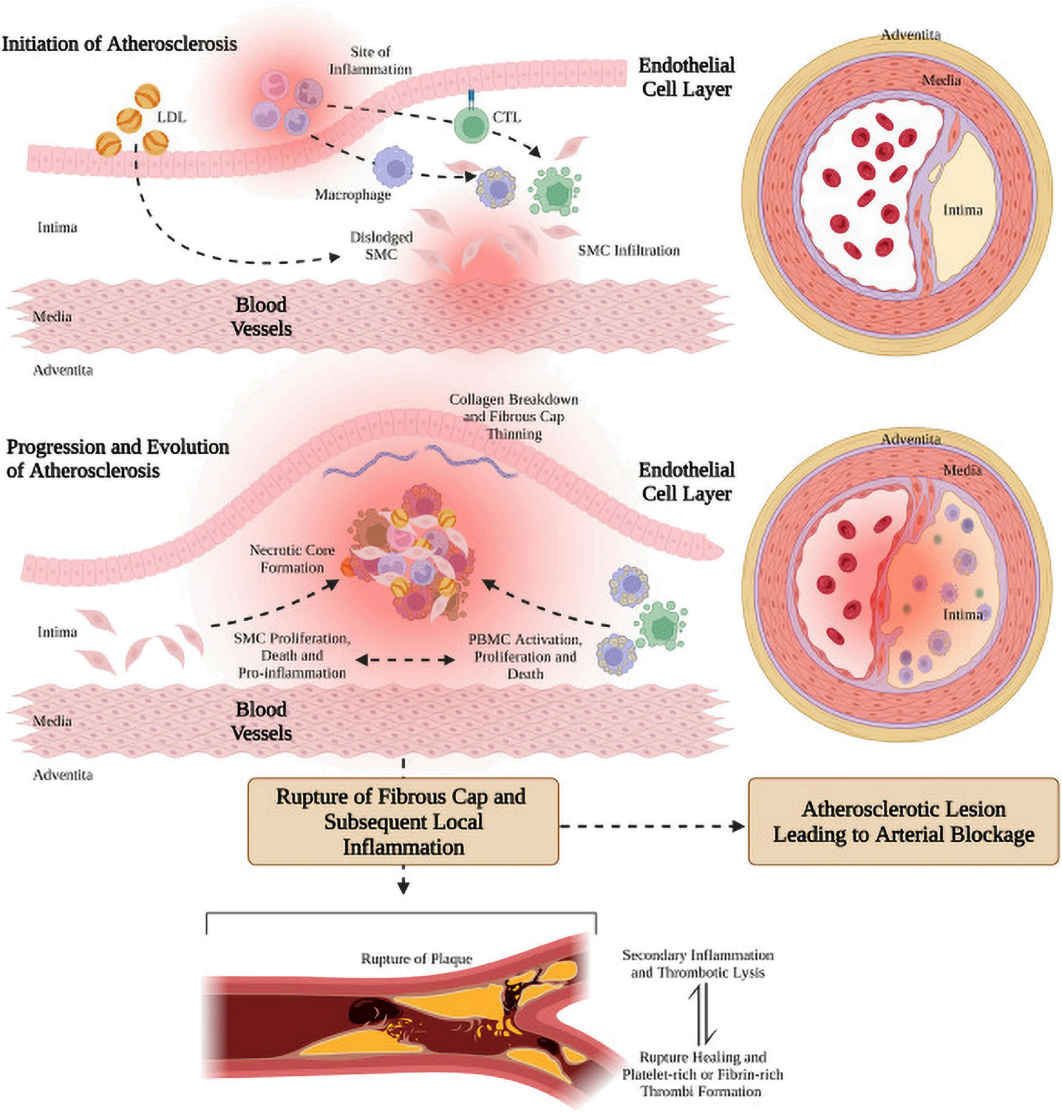
Genesis, progression and evolution of arterial atherosclerotic plaque (Rahagir et al., 2022). Figure generated using BioRender.

The development of vascular AS is shown in Figure 1 (Rahagir et al., 2022). Various risk factors such as chronic hypertension, smoking, and high cholesterol intake, caused damage to the endothelium and induced endothelial cell dysfunction, thereby change in the permeability of the endothelial layer (Libby et al., 2019; Peter, 2021). Dysfunctional endothelial cells secrete a variety of adhesion molecules, which induce the adherence and subsequent accumulation of monocytes or leukocytes in the activated endothelial monolayer and vessel wall. (Gianazza et al., 2020; Mushenkova et al., 2021; Peter, 2021; Mussbacher et al., 2022). Leukocytes or monocytes entering the intima differentiate into macrophages after taking up the deposited oxidized lipids and secrete a large number of inflammatory factors and chemokines, triggering a series of inflammatory responses, and then the macrophages become foam cells (Patel et al., 2015; Tabas and Bornfeldt, 2016). The accumulation of foam cells within vessel walls results in the formation of lipid streaks or AS plaques, which subsequently produce significant amounts of cytokines and chemokines. These molecules further promote the recruitment of monocytes from circulation, ultimately leading to a severe inflammatory response. In addition, stimulated by inflammatory factors, vascular smooth muscle cells (VSMCs) rapidly proliferate and synthesize large amounts of extracellular matrix (ECM) to form a fibrous cap covering the plaque (Lee, 2014; Basatemur et al., 2019; Peter, 2021). With the continuous remodeling and thickening of the vessel wall, the apoptotic VSMCs and foam cells accumulated in the central region of the AS plaque to form a lipid necrotic core, thus forming a severe AS plaque in the arterial vasculature. With the deterioration of AS plaques, plaques become unstable and prone to thrombose (Giovanni and Plinio, 2018), and plaque rupture often leads to acute myocardial infarction and heart failure, resulting in sudden death (Chen et al., 2016).

There are two main clinical treatments for AS. Oral medications, such as statins, which are widely used for AS patients. For the advanced AS (stenosis rate greater than 75%), especially occurred in the coronary arteries, angioplasty and stenting are often used as the first choice. However, the restenosis and thrombosis are still major complications of stent implantation, and it significantly limits the long-term efficacy of stents (Yin et al., 2014). Meanwhile, Oral drug treatment strategies lack targeted therapy capability and have limited drug utilization rates (Wang et al., 2022). To overcome these problems in AS treatment, it is essential to develop more effective and promising therapies. In recent years, with the development of nanomedicine, there is increasing evidence that nanoparticle-based targeting strategies are productive and promising in molecular imaging and treatment of atherosclerosis (Dormont et al., 2018; Pala et al., 2020; Chen et al., 2021; Manners et al., 2022), known as theragnostic nanomedicine (Jun et al., 2015). Nanoparticles could directly penetratethe targeted plaques through injured endothelium or dysfunctional vessels (Lobatto et al., 2011; Lobatto et al., 2015; Wei et al., 2018). The nano agents could be injected intravenously or intraperitoneally, and then the nano agents are cyclically phagocytosed and subsequently translocated to AS lesions by cell recruitment and infiltration (Flogel et al., 2008; Shunsuke et al., 2014; Dou et al., 2017).Furthermore, drugs could be loaded into the nanocarriers that respond to the abnormal microenvironment (ROS, pH, enzymes, and shear stress) of AS lesions (Maruf et al., 2019), thereby increasing the concentration of the drug or imaging molecules at the target lesion site, effectively reducing the side effects of the drug on non-targeted cells, tissues, and organs. In the past decade, significant advancements have been made in the field of therapeutic nanomedicine for AS treatment. Maruf et al. (2019) detailed reported the research progress of responsive nanoagents for AS treatment in recent years, and some nanoagents have been approved for clinical trials. Based on the numerous advantages of nanoagents, this paper provides a comprehensive review of recent research progress on nanoagents targeting AS diagnosis and treatment, including material carriers, target sites, stimuli-responsive nanoagents with different drug release mechanisms under abnormal microenvironments such as ROS, pH, enzymes and shear stress. The aim is to offer insights for the design and preparation of high-performance nanoagents by highlighting their numerous advantages.

## 2 Targets for nanoagents in the treatment of atherosclerosis

**FIGURE 2:**
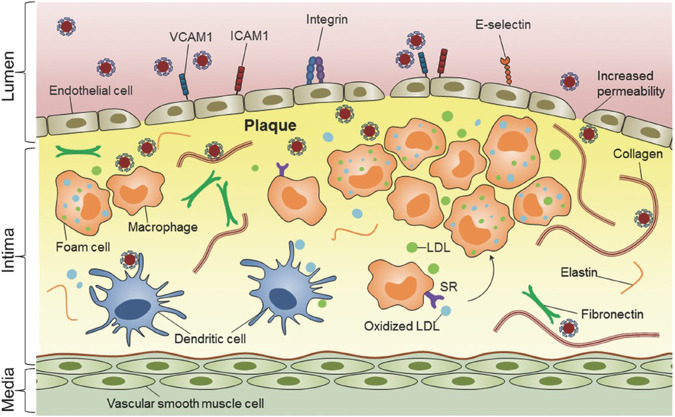
Nanomedicine-based strategies for targeting atherosclerotic plaques (Chan et al., 2018). Copyright 2013, Nature Publishing Group.

The concept nanocarriers entails their swift and precise localization within the AS lesion, akin to a robotic maneuver, followed by targeted drug delivery to the site in need. Hence, it is one direction of developing nano-drug delivery systerm to design a nanocarriers with high specificity and high targeting. Currently, there are two primary approaches for delivering drugs to the lesion site using nanocarriers, one is by enhanced permeability and retention (EPR), and the other is by active targeting (Maruf et al., 2019). The nanoagents could penetrate the incomplete endothelium and accumulate in the AS lesion due to the EPR effect of the AS lesion. The active targeting strategy involves surface modification of nanocarriers, enabling the modified nanoagents to selectively bind to the overexpressed receptors at the AS lesion (Chung, 2016). The active targeting strategy enhances both the aggregation ability and amount of nanoagents at the target lesion site, thereby improving therapeutic efficiency. Nanoagents (Bertrand et al., 2014). For the treatment of AS, there exist numerous specific cells and receptors that can be targeted, as shown in Figure 2. Reseachers have conducted extensive investigations to identify effective targets for treating AS lesions. Scientists have discovered a wide range of specific targets for AS, such as inflammatory vascular endothelial cells, macrophages, extracellular matrix, vascular smooth muscle cells and platelets. Meanwhile, more and more potential targets are discovered by reseachers.

### 2.1 Inflammatory vascular endothelial cells

The vascular endothelium serves as a barrier between the blood and the vascular wall, and it is responsible for maintaining physiological homeostasis of the vasculature under normal conditions (Khaddaj et al., 2017). During the development of AS, the vascular endothelium is impared by continually stimulation from various factors, including immune responses, pathogenic organisms, and hemodynamics dysfunctional (Topper and Gimbrone, 1999; Gimbronejr and García-Cardea, 2013). This leads to an increase in adhesion molecules such as vascular cell adhesion molecule-1 (VCAM-1), intercellular adhesion molecule-1 (ICAM-1), and E-selectin and P-selectin. (Vanhoutte, 1997; Vanhoutte, 2009). These highly expressed adhesion molecules could be used as the potential targets of the nanoagents nanoagents (Figure 2) (Chan et al., 2018).

VCAM-1, also referred to as CD106, is a type I transmembrane protein with a molecular weight of 100–110 kDa. It typically consists of seven C2-type immunoglobulin structural domains and functions as a cell adhesion molecule. VCAM-1 is expressed in both early and advanced AS lesions, indicating its potential as a biomarker for vascular inflammation and endothelial cell dysfunction (Galkina and Ley, 2007; Fotis et al., 2012). Currently, antibodies to specific targeting peptides against VCAM-1 are usually modified on the surface of nanoparticles to target the inflammatory endothelium. Tsourkas et al. (2005) covalently attached VCAM-1 antibody onto iron oxide nanoparticles and labeled them with the near-infrared fluorescent dye Cy5.5, enabling detection of VCAM-1 expression level on endothelial cells and labeling of activated endothelial cells.

**FIGURE 3:**
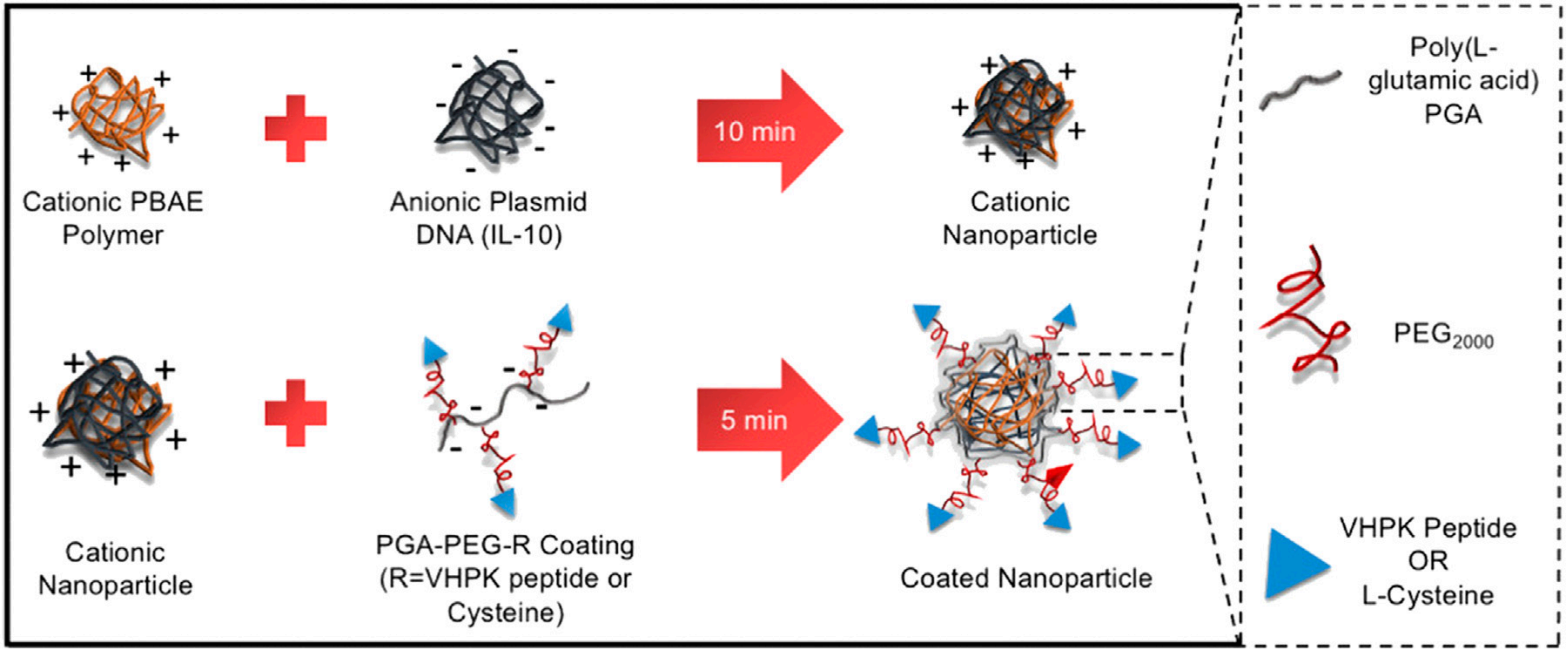
Synthesis of VCAM-1-targeted nanoparticles (NP- VHPK) (Distasio et al., 2020).

Sun et al. (2016) coupled a VCAM-1 targeting peptide and miRNA inhibitor (anti-miR-712) to a DNA vector with complementary sequences modified on the surface of gold nanospheres and selectively delivered anti-miR-712 to mouse aortic endothelial cells with elevated VCAM-1 expression to inhibit AS plaque formation. Distasio et al. (2020) synthesized a polyglutamic acid (PGA) coating (PGA-PEG-VHPK) containing polyethylene glycol (PEG) and VCAM-1 specific targeting peptide (VHPK), and mixed polyβ-amino ester (PBAE) and DNA plasmid (pDNA) solution to prepare nanoparticles (NP), and then a targeting coating was applied to obtain a VCAM-1 targeting nanoparticle (NP-VHPK) (Figure 3). The interaction between the NPs and the VCAM-1 receptor was studied by surface plasmon resonance and imaging (SPRi) under different physiological conditions. The findings revealed that transfection of inflammatory endothelial cells under static and dynamic flow conditions resulted in a significantly higher internalization rate of NP-VHPK compared to non-targeted controls (80% vs. 30% positive cells). In areas of AS plaque in the mouse aorta and aortic sinus, NP- VHPK rapidly bounded to inflammatory endothelial cells with high VCAM-1 expression. Additionally, the NP-VHPK could be localization in the thoracic region adjacent to the heart and the blood vessels in the atherosclerostic mouse model through jection. These results suggested that surface modification with specific antibodys, peptides or other targeting agents could significantly enhance targeting efficiency and bioavailability. In addition, VCAM-1, ICAM-1 and selectin, which are highly expressed in the inflammatory endothelium of AS lesions, may serve as potential targets for specifically targeting (Bhowmick et al., 2012; Park et al., 2008; Chan et al., 2018).

### 2.2 Macrophages

**FIGURE 4:**
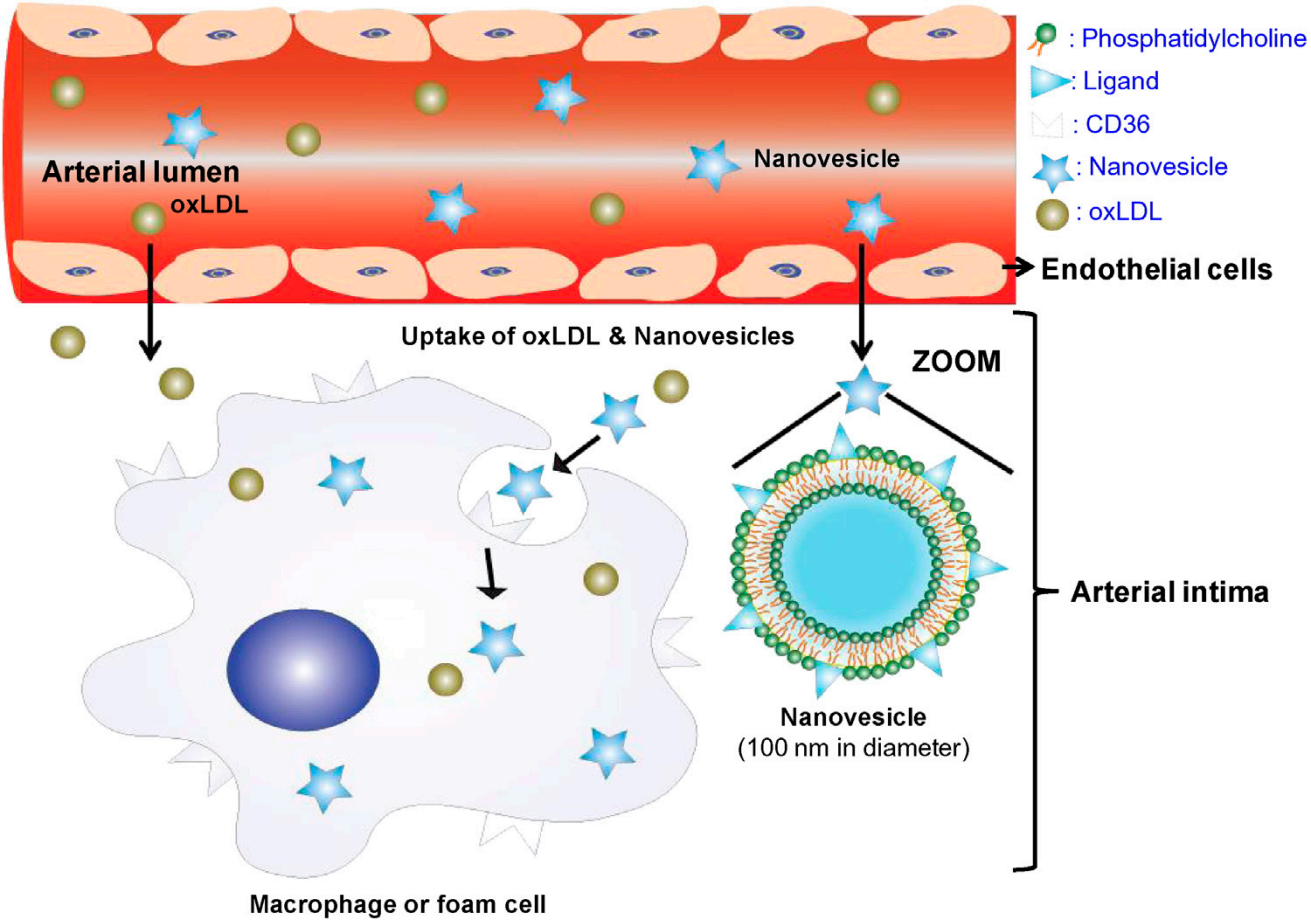
The composition, structure and targeting mechanism of CD36-targeted liposome nanoparticles (Nie et al., 2015).

Macrophages are typically located in the tissues and differentiated from the monocytes in the blood. Macrophages, as we foam cells drived from macrophage after phagocytized large amounts of lipids, play an important role in all stages of AS lesion development, from formation to plaque rupture (Wu et al., 2018; Moroni et al., 2019). By designing nanoparticles that carry lipid-lowering drugs, anticoagulant drugs, siRNA, DNA plasmids and other therapeutic agents to target macrophages, the development of AS can be effectively inhibited (Sato et al., 2014; Dipti et al., 2016). In AS lesions, macrophages adhere to and accumulate in the vessel wall through scavenger receptors on their surface, such as CD36, lectin-like oxidized low-density lipoprotein receptor-1 (LOX-1), and macrophage scavenger receptor-1 (MSR-1) (Reyk and Jessup, 1999; Murray and Wynn, 2011). These transmembrane glycoproteins exhibit a broad distribution *in vivo* and bind to diverse ligands. As a scavenger receptor, CD36 plays crucial roles in both lipid metabolism and the adhesion of anionic biomolecules. The findings suggest that CD36 plays a crucial role in lipid uptake and the development of atherogenesis (Febbraio et al., 2000; Kathryn and Mason, 2006), making it a common target for macrophage-directed therapy in AS lesions. For instance, the GHRPs protein family was employed to specifically target macrophages that exhibit high levels of CD36 expression in AS lesions (Demers et al., 2004). Nie et al. (2015) prepared liposome nanoparticles from phosphatidyl choline and KOdiA-PC (Figure 4). The liposomal nanoparticles were intravenously injected into low-density lipoprotein receptor (LDLR)-deficient mice, and it was found that these nanoparticles exhibited a remarkable ability to selectively target and bind to macrophages in AS lesions by binding specifically to CD36 receptors. The CD36-targeted nanoparticles were able to bind specifically to macrophages, resulting in a reduction of macrophage deposition within the AS lesion. This was achieved with small interfering RNA (siRNA) loaded into the nanoparticles, which effectively silenced CD36 expression in macrophages. LOX-1 plays a pivotal role in the pathogenesis of AS by acting as a major receptor of oxidized low-density lipoprotein (oxLDL), which contributes to endothelial cell dysfunction, monocyte adhesion, VSMCs migration and foam cell formation (Tian et al., 2019). Therefore, targeting LOX-1 may represent an effective therapeutic strategy for AS. In a study by Song et al. (2014), LOX-1 antibodies were conjugated onto the surface of superparamagnetic nanoparticles, resulting in higher binding affinity and increased uptake by RAW 264.7 macrophages compared to non-targeted modified nanoparticles. Following intravenous administration of the nanoparticle in ApoE^−/−^ mice, magnetic resonance imaging (MRI) revealed a significant enhancement signal at AS lesions, particularly in regions enriched with macrophage/foam cells. Additionally, MSR-1, an important scavenger receptor located on the surface of macrophages involved in oxLDL uptake by these cells (Winther et al., 2000). Modified nanoparticles with MSR-1 peptide-like ligands or antibodies on their surface could bind to macrophages in AS lesions, facilitating targeted delivery to these cells (Segers et al., 2013). In addition, the nanoparticles can specifically target macrophages through endocytosis mediated by glycosylated receptors such as mannose receptor (MR), galactose C-type lectin 1/2 (Mgl1/2, and folate receptor (FR) expressed on the surface of macrophages (Mohammadi et al., 2016; Alvarado-Vazquez et al., 2017; Daphne et al., 2019). The utilization of folic acid-modified radioactive material has been demonstrated to enable selective targeting of FR-β-positive macrophages for noninvasive imaging of vascular inflammation (Padilla et al., 2014).

During the development of AS, monocytes infiltrate the endothelium and differentiate into macrophages bytaking up oxidized lipids deposited within the arterial wall. These macrophages then secrete large amounts of inflammatory factors (e.g., TNF-α, IL-6, IL-1β, etc.), triggering a series of inflammatory responses that promote further progression of atherosclerosis. The production and release of inflammatory cytokines are effectively regulated by either introducing anti-inflammatory agents into the macrophage cytoplasm or by reducing the expression of inflammatory genes through RNA interference (siRNA) (Dinarello, 2010). Therefore, macrophages in AS lesions target not only inflammatory factors but also members of nuclear transcription factors (NF-κB) signaling pathways involved in promoting inflammatory transduction (Tornatore et al., 2012). For example, Wang et al. (2018) covalently conjugated antioxidants to the cyclic polysaccharide β-cyclodextrin (β-CD) to obtain antioxidant nanoparticles and found that the nanoparticles were internalized by macrophages and VSMCs efficiently and rapidly. Then, the inflammation and apoptosis of macrophage induced by inflammatory factorswere mitigated through the inhibition of pro-inflammatory factors such as TNF- α, IL-1β and MCP-1. Meanwhile, foam cell formation and the development of AS in ApoE ^−/−^ mice were effectively inhibited. Zhang et al. (2021) employed baicalein to target and inhibit the downstream signaling of MAPKs/NF-κB, effectively suppressing the production of pro-inflammatory factors such as IL-6, TNF-α and PAI-1.

### 2.3 Extracellular matrix

The extracellular matrix (ECM) is a complex network of proteins and polysaccharide macromolecules secreted by cells in the extracellular mesenchyme, which interconnects tissue structures and orchestrates tissue development as well as cellular physiological activities. Studies have demonstrated the crucial role of ECM in the development of AS and the formation of fibrous cap (Adiguzel et al., 2009; Lee, 2014; Basatemur et al., 2019), as well as its potential contribution to plaque destabilization. Hence, ECM also could be used as a common target for the treatment of AS.

Collagen, a major component of the ECM, influences the strength and integrity of the fibrous cap in the development of AS, and it regulates cellular responses through specific receptors and signaling pathways (Adiguzel et al., 2009). Collagen is commonly target in the ECM. Chan et al. (2010) have identified peptide sequence that exhibits high affinity for type IV collagen (Col IV) through phage display investigations. This protein is highly expressed at sites of vascular injury, and given that more than 50% of the lesion basement membrane is composed of Col IV (Kalluri, 2003). Therefore, modifying collagen-targeting peptides on the surface of nanoparticles is an effective strategy for targeting atherosclerotic plaques. As an illustration, Kamaly et al. (2016) used nanoprecipitation to induce the self-assembly of a double-block polymer to form a collagen IV-targeting nanoparticle (Col IV IL-10 NP22) containing the anti-inflammatory factor interleukin-10 (IL-10), and these NPs have anti-inflammatory effects on macrophages both *in vivo* and *in vitro*. The particles have the ability to prevent vulnerable plaque formation by increasing the thickness of fibrous cap and reducing necrotic cores. Meyers et al. (2017) immobilized collagen-targeting peptides onto the surface of gold nanoparticles, resulting in the production of a collagen IV-targeting gold nanoparticle (T-AuNP) that specifically bonded to injured vascular sites.

In addition to collagen, other proteins in the ECM, polysaccharides, and other macromolecules observed on the surface or inside the plaque, which are associated with the progression of AS (Bini, 1989; Hultgardh-Nilsson et al., 2015; O'Brien et al., 2005), also serveas targets for the diagnosis and treatment of AS. Yoo et al. (2016) developed one kind of amphiphilic microparticles (PAMs) that incorporate fibrin-binding peptides, enabling targeted imaging of AS plaques using both MRI and NIRF techniques. Dipti et al. (2017) designed an emulsion agent with ω-3-fatty acid flaxseed oil coated with 17-βE and modified CREKA targeting peptide on its surface to specifically bind to fibrin clots. And, *in vivo* experiments demonstrated that the pro-inflammatory factors could be downregulated by the agent in ApoE^−/−^ miceand the lesion area also be reduced while possessing good biocompatibility.

### 2.4 Vascular smooth muscle cells

**FIGURE 5:**
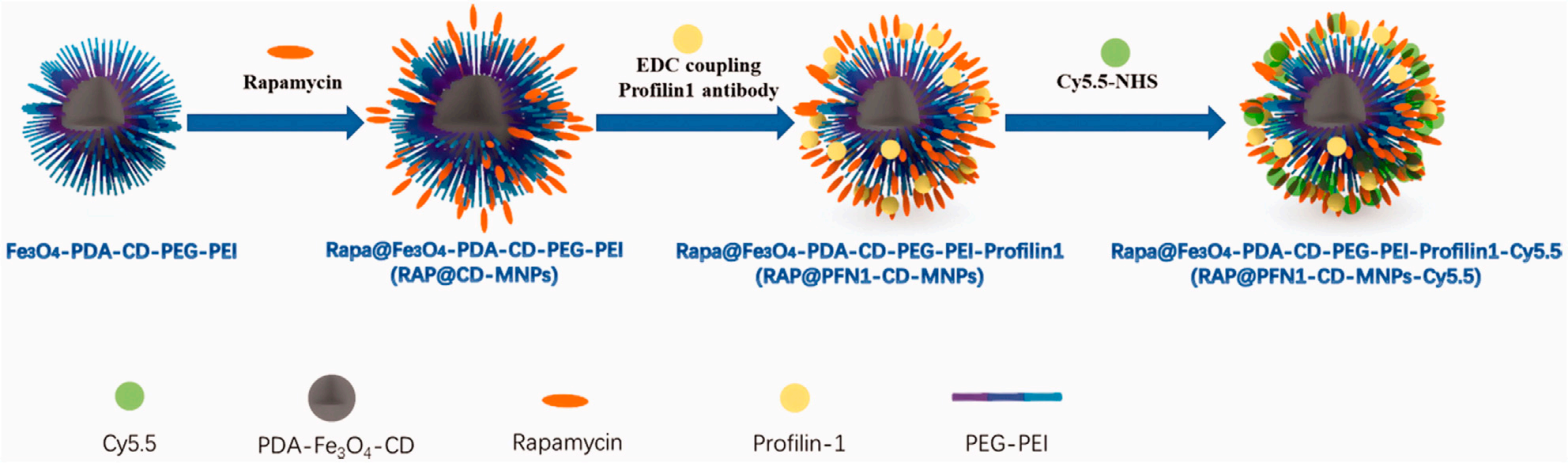
Schematic diagram of RAP@PFN1-CD-MNPs nanoparticles (Zhang et al., 2020).

Vascular smooth muscle cells (VSMCs) are the predominant cellular constituents of the vessel wall, responsible for maintaining vascular tone and structural integrity. Normal VSMCs are primarily located in the media layer of blood vessels and predominantly express contractile components such as smooth muscle myosin heavy chain and α-smooth muscle actin. In contrast, in AS lesions, the intima also harbors VSMCs, which exhibit a high proliferation index and predominantly synthesize extracellular mesenchyme, proteases and cytokines, while expressing limited contractile components (Br, 2004). Studies have demonstrated that VSMCs undergo a phenotypic switch from contractile to synthetic in response to various factors, including injury and lipid exposure (Pidkovka et al., 2007). Subsequently, the contractile capacity of VSMCs diminishes and they become responsive to various stimuli such as fibroblast growth factor (FGF), platelet-derived growth factor (PDGF), etc., which are released in response to vascular injury. This leads to the migration and proliferation of VSMCs, thereby promoting further development of AS.Studies have indicated that the proliferation and migration of VSMCs are closely linked to their expression of pluripotency factors, such as Oct4/Pou5f1 and KLF4 (Cherepanova et al., 2016; Majesky et al., 2017). Therefore, regulating the expression of these factors may offer a promising approach for treating atherosclerosis. Currently, there is a dearth of research on the treatment of AS with VSMCs. However, given their pivotal role in the pathogenesis and progression of AS as well as their distinctive phenotypic characteristics from normal VSMCs, targeting VSMCs may hold promise for therapeutic intervention in AS. Zhang et al. (2020) combined the VSMC- targeting profilin-1 antibody (PFN1) with the anti-inflammatory drug rapamycin (RAP) and loaded it on a complex of superparamagnetic iron oxide and pH-sensitive cyclodextrin to create an integrated therapeutic and MRI imaging nanoparticle (RAP@PFN1-CD-MNPs) (Figure 5). The PFN1-CD-MNPs were demonstrated to effectively bind to VSMCs for targeted localization of AS lesions, followed by rapid drugrelease in their micro-acidic environment, thereby mitigating the progression of atherosclerosis.

### 2.5 Platelets

Platelets, anucleated blood cells produced by megakaryocytes in the bone marrow and lungs, possess coagulation and hemostatic functions that are essential for maintaining physiological homeostasis and repairing vascular damage Carsten (2018). In addition, platelets could adhere to various cells and factors, thereby participating in a series of cascading inflammatory responses that promote the formation of AS. Studies have also found an elevation in platelet adhesion to the vessel wall at the site of AS lesion. (Lievens and Hundelshausen, 2011; Christian and Steffen, 2012; Eduardo et al., 2013). Therefore, platelets also could be a potential target for targeting AS. For example, Jacobin-Valat et al. (2015) prepared a multifunctional superparamagnetic iron oxide nanoparticle (VUSPIO) that were surface-modified with a recombinant antibody (rIgG4 TEG4), enabling them to bind to activated platelets. Bachelet-Violette et al. (2014) modified superparamagnetic iron oxide nanoparticles with a sulfated polysaccharide that could specifically bind to P-selectin. The functionallized nanoparticles exhibited specific binding affinity towards activated platelets in the bloodstream, rendering them suitable for MRI imaging of AS lesions. However, nanoparticles targeting P-selectin may be potentially limited due to the expression of P-selectin on inflammatory endothelial cells, which challenges the specificity of these nanoparticles binding to platelets. Therefore, further investigations are warranted to validate the reliability of platelets as a viable target for both diagnosis and treatment of AS.

### 2.6 Other targets

Non-coding RNAs (ncRNAs) are emerging as crucial regulators of cellular function and disease progression (Poller et al., 2018; Yang et al., 2020), with a key role in cardiovascular disease (Liu et al., 2018; Tang et al., 2018; Liu et al., 2020; Mengdie et al., 2020). Among ncRNAs, micrRNAs (miRNAs) are one of the most extensively studied and widely researched with a length ranging from 20 to 25 nucleotides. MiRNAs participate in a diverse range of physiological processes and pathological conditions primarily by exerting post-transcriptional repression on their target mRNAs, thereby impeding the translation of proteins encoded by the genetic information carried within these transcripts. (Aryal and Suárez, 2019; Mori et al., 2019). In the AS lesions, miRNAs generally regulate the expression of cell adhesion molecules to modulate inflammatory responses through two distinct mechanisms, transcriptional regulation of the pro-inflammatory NF-κB signaling pathway and direct targeting. Different miRNAs are responsible for regulating different pro-inflammatory NF-κB signaling pathways Zhong et al. (2018). Additionally, miRNAs have multiple functions in regulating vascular cell homeostasis, participating in lipoprotein secretion and metabolism, and immune responses, and play a key role in the progression, modulation, and regulation of each stage of AS (Feinberg and Moore, 2016; Shoeibi, 2020). MiRNAs have emerged as a novel class of therapeutic targets for AS disease in recent years (Yang et al., 2017; Wang et al., 2020; Zong et al., 2021). Apelin is an endogenous ligand for the angiotensin II type 1 receptor-related protein APJ, which exhibits a wide distribution in various organs and tissues of both animals and humans. Apelin exerts various physiological effects on the cardiovascular, immune, nervous and other systems by binding to the APJ receptor. It is involved in pathophysiological processes such as inflammatory, oxidative stress, hormone secretion regulation, vascular tone maintenance and myocardial contractility regulation (Mughal and O’Rourke, 2018). It also exhibits antagonistic effects with the renin-angiotensin system and participates in the regulation of the stress response. Therefore, APJ receptor agonists or antagonists are anticipated to emerge as novel targets for the diagnosis and treatment of AS by nanoagents.

## 3 Stimulus-responsive nanoagents for AS treatment

**FIGURE 6:**
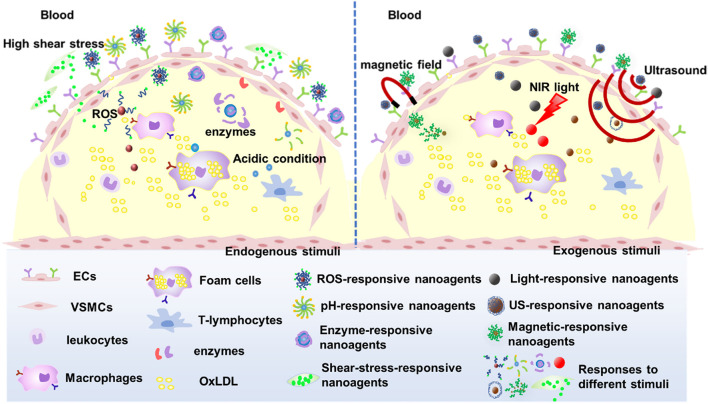
Smart nanoagents for targeted treatment of atherosclerosis.

The efficient delivery and intelligence targeting of drugs are also critical for AS treatment. Hence, nano-agents have been designed to respond to endogenous stimuli based on the abnormal microenvironment at the AS lesion site such as micro-acidity, high shear stress due to accelerated blood flow, overexpressed enzymes and reactive oxygen species (ROS), among others. The small molecule drugs were encapsulated within the nanocarriers through chemical or physical reactions, and these nanocarriers maintain their structural integrity in normal tissues. Upon arrival at the lesion site, the nanocarriers undergo depolymerization triggered by specific stimuli and subsequently release drugs, thus mitigating systemic toxicities associated with small molecule therapeutics. In addition, nanoagents can be intelligently designed to respond to exogenous stimuli such as light, magnetic fields, or multiple stimuli to enhance the efficacy of nanoagents for AS (Figure 6).

### 3.1 ROS responses

**FIGURE 7:**
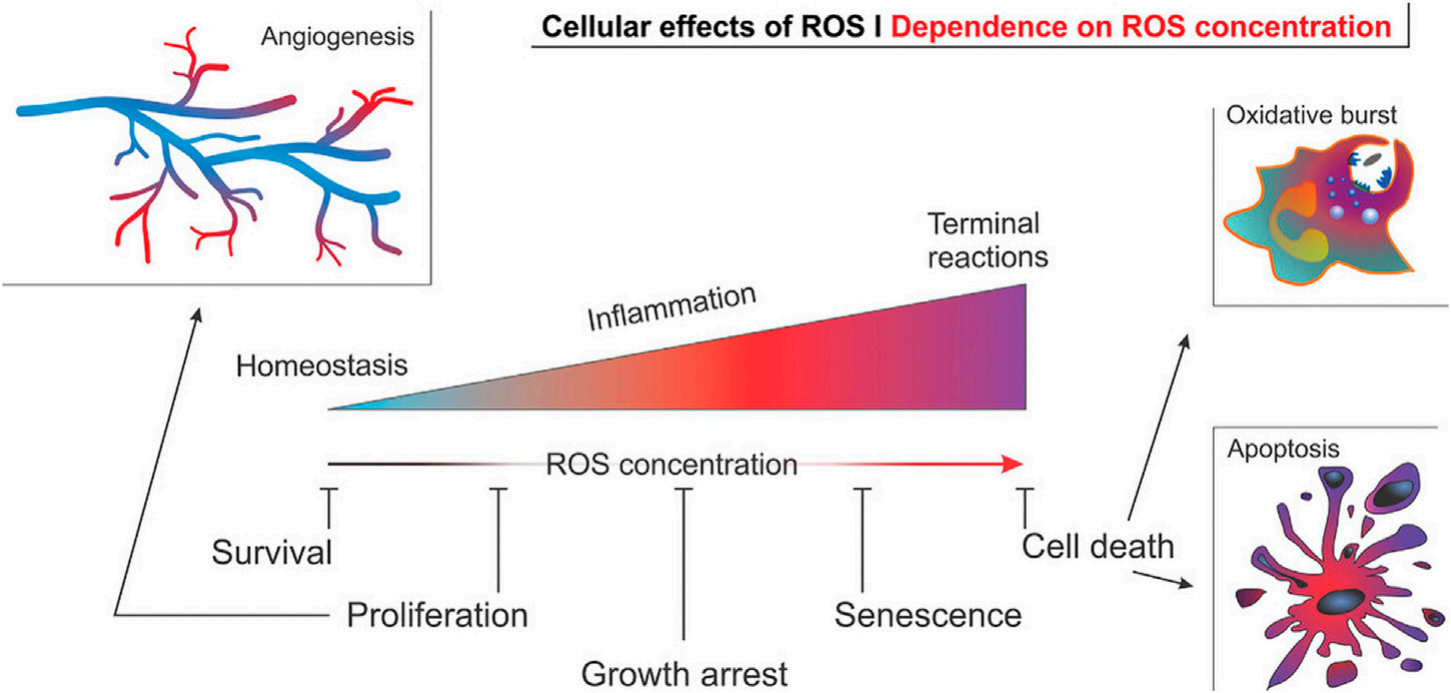
Effect of ROS concentration on the physiological activity of cells in angiogenesis (El-Mohtadi et al., 2018).

ROS is a collective term for a class of small reactive molecules that are continuously produced and utilized in all living systems. They play a key role in maintaining self-homeostasis of vascular tissue and regulating a variety of cellular functions (El-Mohtadi et al., 2018). ROS is primarily comprised of superoxide, hydroxyl radicals, hydrogen peroxide (H_2_O_2_), peroxynitrite and hypochlorite (Yuliya et al., 2015; Nowak et al., 2017). ROS levels are a key factor in the pathogenesis of AS and angiogenesis, as cellular physiological activities are intricately linked to ROS concentrations (El-Mohtadi et al., 2018) (Figure 7).

High ROS levels lead to an increase in oxidized lipoproteins (Ox-LDL), endothelial dysfunction, DNA damage, leukocyte migration and differentiation, proliferation of VSMCs, and elevated MMPs. According to the ROS high levels environment in AS diseased tissues, researchers have explored various ROS-responsive drug carriers. These carriers are typically constructed using polymers containing sulfur, selenium or tellurium, phenylboronic acid ester and co-administered photosensitizer ROS sensitive structures.

#### 3.1.1 ROS response of sulfur, selenium or tellurium polymers

**FIGURE 8:**
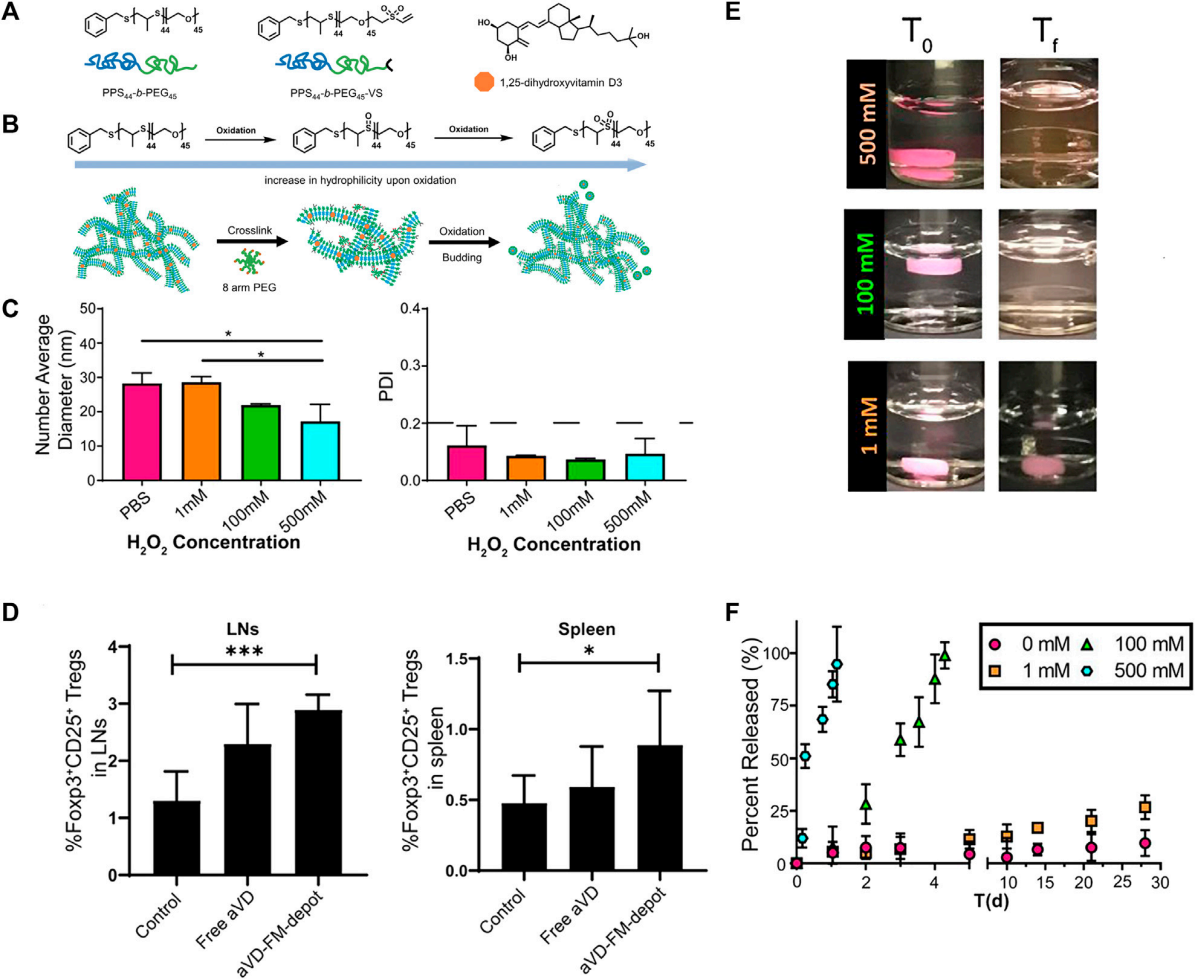
ROS-responsive filamentous hydrogels (FM-depots). **(A)** Structure of PEG-b-PPS block copolymer and 1,25-dihydroxyvitamin D3 (aVD). **(B)** Schematic of morphological transformation of FM-depots. **(C)** Average diameters and PDI of aVD-FM at different H_2_O_2_ concentrations. **(D)** aVD-loaded FM-depots elicit Treg responses in lymph nodes and spleen of ApoE^−/−^ mice. **(E)** Pictures of aVD-loaded FM-depots in PBS before and after addition of different concentrations of H_2_O_2_. **(F)** *In vitro* drug release kinetics of aVD-loaded oxidized at different H_2_O_2_ concentrations for 30 days.

Polymers containing sulfur, selenium or tellurium exhibit similar properties due to the homology of these non-metallic elements, which can undergo both oxidation and reduction reactions. Hence, polymers containing sulfur, selenium or tellurium are susceptible to oxidation and degradation. Therefore, nanocarriers made from such polymers respond to the ROS environment by releasing drugs. Wang et al. (2015) obtained ultra-sensitive ROS-sensitive assemblies using tellurium-containing molecules self-assembled with phospholipids. Scott et al. (Karabin et al., 2018; Yi et al., 2020) developed an ROS-responsive filamentous hydrogel (FM-depots) containing poly (ethylene glycol)-block-poly (propylene sulfide) copolymers (PEG-b-PPS), capped with methyl vinyl sulfone (Figure 8A) and loaded with the anti-inflammatory agent 1,25-dihydroxyvitamin D3 (aVD). The hydrogel was injectable and sustainably delivered anti-inflammatory drugs through thiol cross-linking with 8-arm PEG. The main component of this hydrogel was These aVD-loaded filamentous fibers undergoed morphological transformation during oxidation of H_2_O_2_, resulting in the release of spherical drug-loaded micelles (Figure 8B), whose diameter and PDI were less affected by the H_2_O_2_ concentration (Figure 8C), but the drugs release efficiency depended on the oxidant concentration (Figures 8E,F). So that this drug-loaded hydrogel sustainable released drugs in oxidation condition. After subcutaneous injection into ApoE^−/−^ mice, aVD-loaded FM-depots maintained high levels of Foxp^3+^ treg in lymphoid organs and AS lesions for several weeks (Figure 8D).

**FIGURE 9:**
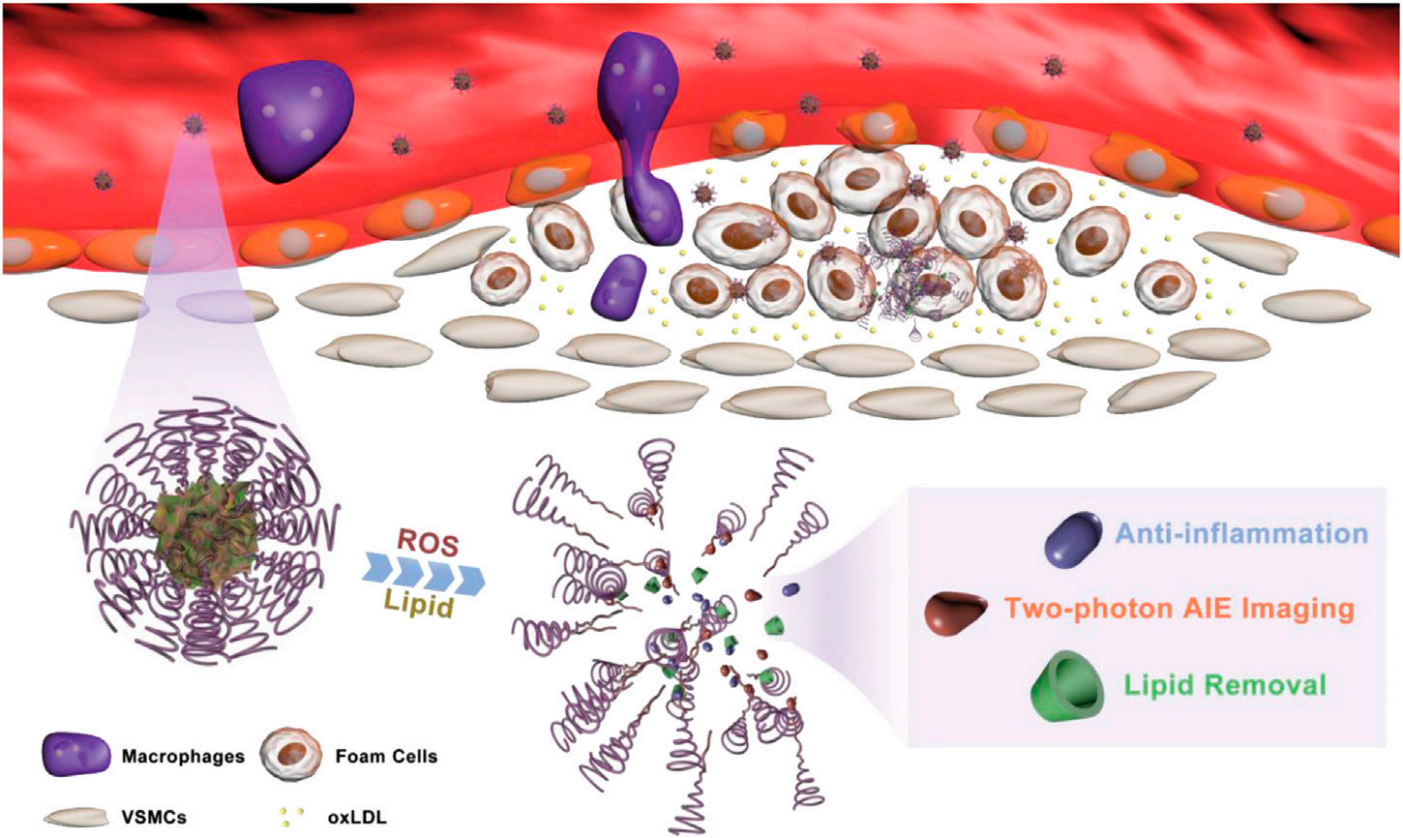
Schematic of atherosclerotic plaque identification and inhibition by TPCDP@PMM (Ma et al., 2020).

Ma et al. (2020) combined a two-photon aggregation-induced emission (AIE) active fluorophore (TP) with β-cyclodextrin (CD) with ROS responsive bond and carried prednisolone (Pred) into its inner lumen via molecular interactions, and then a diagnostic-therapeutic composite two-photon fluorophore-cyclodextrin/prednisolone complex (TPCDP) was constructed. The TPCDP consisted of a ROS-sensitive copolymer poly (2-methylthioethanol methacrylate)—poly (2-methacryloyloxyethyl phosphorylcholine) (PMM) wrapped into nano-micelles (TPCDP@PMM), and the TPCDP was enriched in the damaged vascular endothelium by EPR effect. Due to the relatively strong interaction of lipids with CD, micelles were disrupted by the activation of locally overexpressed ROS and abundant lipids, and TPCDP were further dissociated with the release of Pred, resulting in anti-inflammatory activity and lipid clearance to inhibit AS (Figure 9). In addition, TPCDP@PMM with TP labeling indicated that two-photon AIE imaging was also useful for the identification of AS and it could be used for the diagnosis of AS.

#### 3.1.2 Phenylboronic acid ester polymers ROS response

**FIGURE 10:**
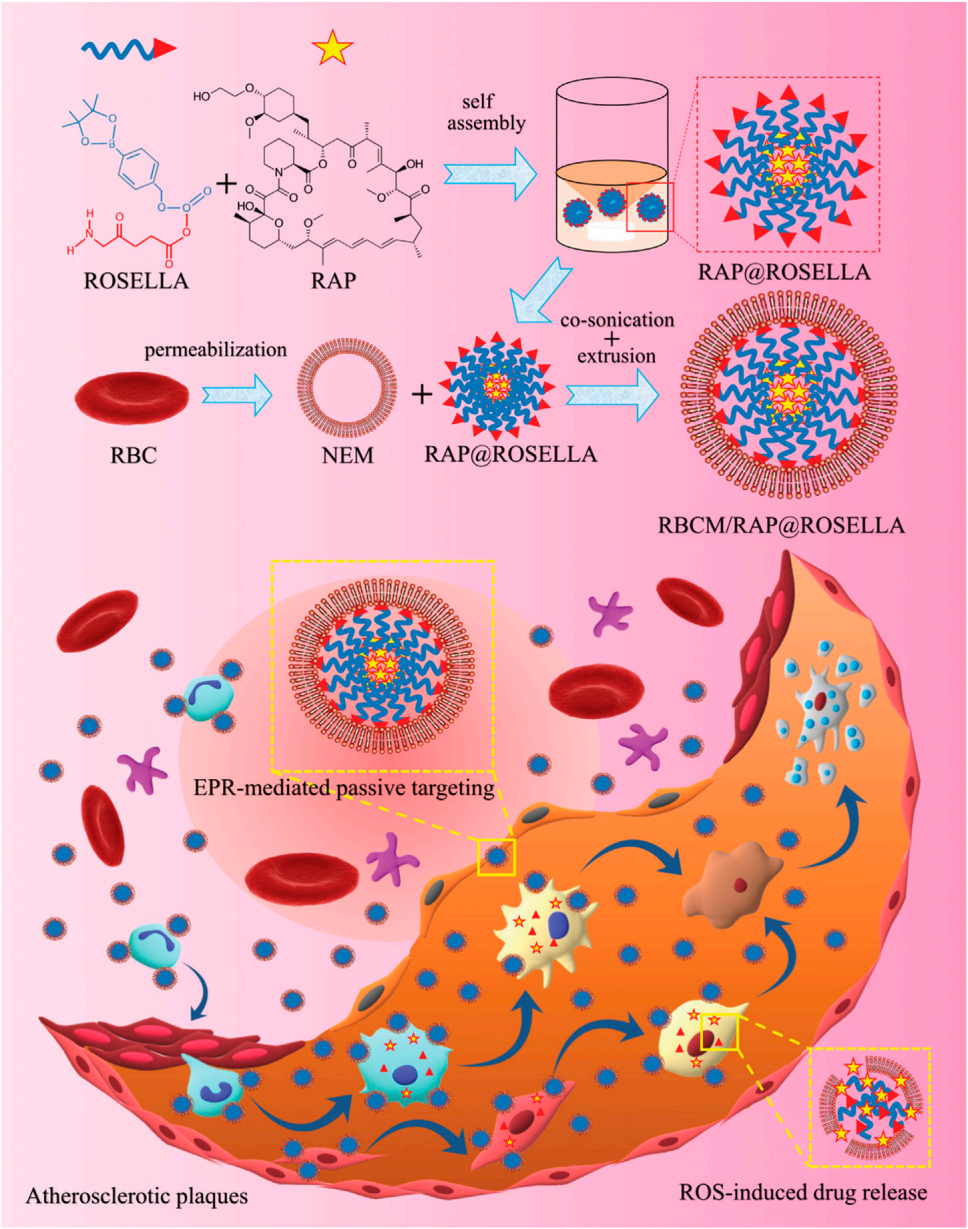
Synthetic of RBCM/RAP@ROSELLA and schematic diagram of RBCM/RAP@ROSELLA targeting of atherosclerotic plaques (Maruf et al., 2021).

The phenylboronic acid moiety is a group sensitive to oxidative stress, which can be oxidized into phenols and boronic acids under H_2_O_2_ conditions or undergoes quinone structural rearrangement. Therefore, the use of phenylboronic acid ester polymers as drug carriers is prone to chemical bond breakage caused by oxidation in a high ROS environment, resulting in drug release. Maruf et al. (2021) utilized 5-aminolevulinic acid (ALA) and phenylboronic acid to construct ROS-responsive nanoparticles (RAP@ROSELLA) via self-assembly, which were then loaded with rapamycin (RAP). Subsequently, the nanocellular membrane (NEM) was enveloped around the nanoparticles to yield bionanobionic nanoagents (RBCM/RAP@ROSELLA) (Figure 10). These nanoagents were able to escape the biological barrier, enriched at the AS lesion site through the EPR effect and released the drugs due to its high level of ROS environment. *In vitro* experiments demonstrated that RBCM/RAP@ROSELLA exhibited superior inhibitory effects on the proliferation of macrophages and VSMCs. Zhao et al. (2020) inked tirofiban to phenyl borate ester bonds and dextran through coupling, and subsequently incorporated it into the erythrocyte membrane. Then, the fibronectin-targeting peptide CREKA was modified onto the membrane surface to form a ROS response (T-RBC-DTC NPs) nanoparticles. In a mouse FeCl_3_ thrombosis model, T-RBC-DTC NPs were abundantly enriched at the site of damaged carotid arteries and released drugs via ROS response with significant antithrombotic effects.

#### 3.1.3 Combined photosensitizer ROS response

**FIGURE 11:**
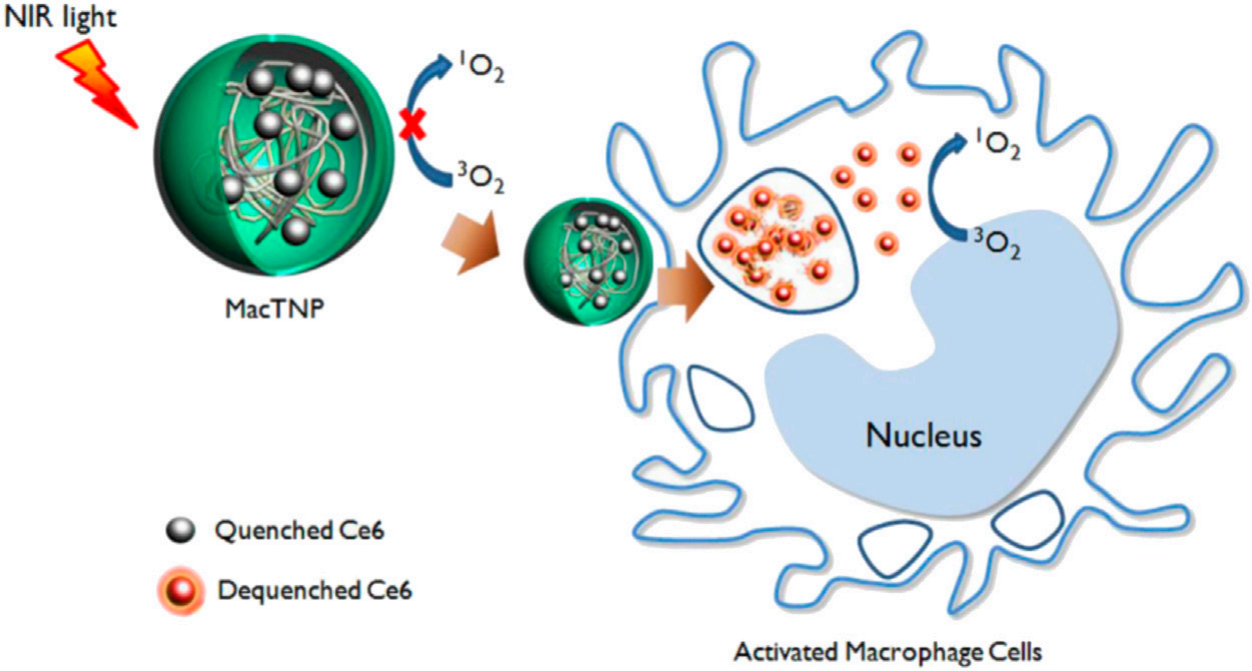
Schematic diagram of fluorescence imaging of MacTNPs-targeted macrophages and subsequent photodynamic therapy (Kim et al., 2014).

Certain O_2_-sensitive polymers, when coupled with photosensitizers and irradiated at specific wavelengths, generate O_2_ and undergo oxidation or cleavage to release drugs. Therefore, these photosensitizer-polymer conjugates are also utilized as ROS-responsive nanocarriers. For example, Kim et al. (2014) prepared ROS-responsive macrophage-targeted therapeutic nanoparticles (MacTNPs) by coupling the photosensitizer chlorin e6 (Ce6) with hyaluronic acid (HA). The HA coating on the surface of MacTNPs protected the NPs from NIR activation, thereby preventing leakage of photosensitized substances notform the MacTNPs particles. When MacTNPs contact with the activated macrophages, the HA on the surface of NPs was degraded by overexpressed ROS and released photosensitive substances. These substances could respond to NIR and emitted fluorescence, to precise the position of AS lesions (Figure 11). The photosensitized substances induced 66% of macrophage death upon light exposure by generating singlet oxygen. It had also been shown that photodynamic therapy (PDT)-mediated cells autophagy was superior to cell necrosis. Therefore, PDT treatment has the potential to stabilize or inhibit the development of AS by inhibiting and reducing the number of macrophages.

### 3.2 pH responses

The acidic cellular microenvironment at the site of inflammation is a well-established fact. Macrophages present at the AS plaque site phagocytosing large amount of Ox-LDL, leading to significant accumulation of lactic acid and further aggravating the local acidity (Parathath et al., 2013; Zhao and Herrington, 2016). Katariina et al. (2015) utilized two pH-sensitive dyes to determine the pH levels within atherosclerotic plaques, revealing that human atherosclerotic plaques exhibited a pH range of 6.5–8.5 while rabbit was 5.5–7.5. The pH of lysosomes in macrophages was measured at 4.7–4.8. The weakly acidic microenvironment (pH 6.0–6.8) of atherosclerotic lesions and the acidic environment (pH below 5.0) of macrophages lysosomes (Ohkuma and Poole, 1978) can be exploited for precise atherogenic response therapy. Currently, various types of pH-responsive nanoagents have been designed and constructed for the treatment of AS, including those initiated by covalent bonds, intermolecular forces, and physical structures.

#### 3.2.1 Covalent bond-initiated

Surface functionalization of nanomaterials so that they are connected to drug molecules by covalent bonds is the most common way to obtain smart nanoagents, and this type of nanoagents exhibits high stability and effectively prevents the premature release of drugs under physiological conditions. The therapeutic effect is better if the drug relates to covalent bonds with pH responsiveness, such as acetal (ketone), amine bond, ligand bond, hydrazone bond, etc.

**FIGURE 12:**
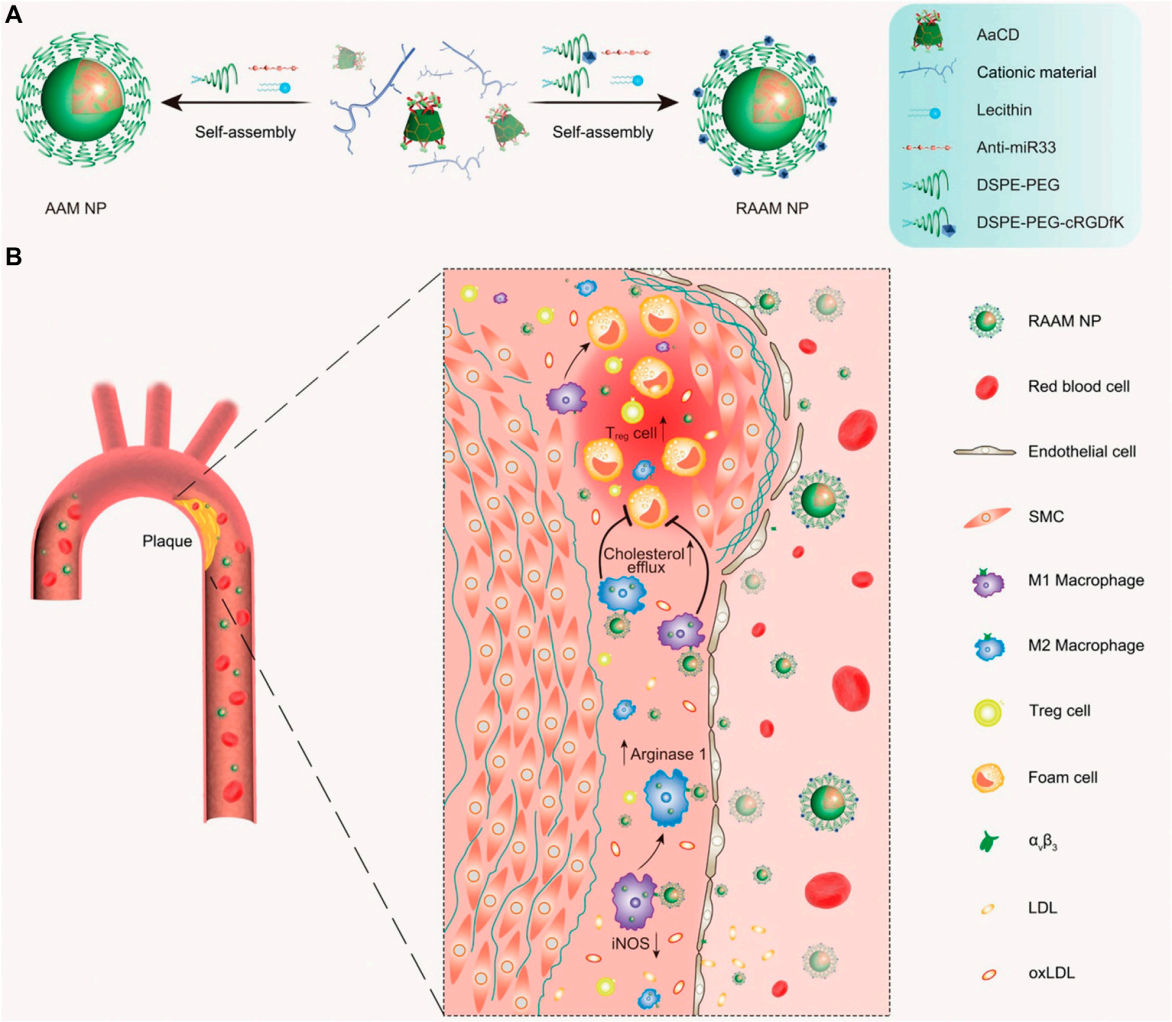
Schematic representation of the synthesis and targeting of pH-responsive anti-miR33 nanoparticles for the treatment of atherosclerosis. **(A)** The composition and preparation of designed anti-miR33 nanotherapies AAM and RAAM. **(B)** Sketch showing targeted treatment of atherosclerosis with the active targeting nanotherapy RAAM by simultaneously regulating reverse cholesterol transport and lesional immune responses (Li et al., 2020).

**FIGURE 13:**
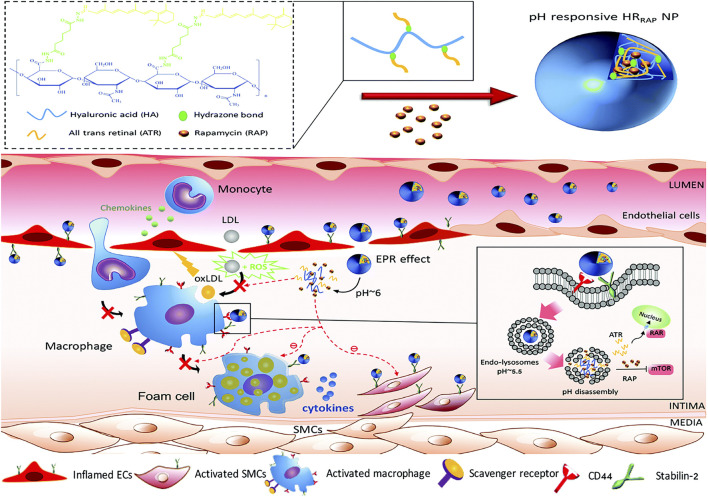
Illustration of the formation of pH-sensitive HR_RAP_NP and schematic illustration of HR_RAP_NPs accumulation at the atherosclerotic plaque through the combination of EPR effect and nanoparticles-HA receptors interaction (Nihad et al., 2022).

Li et al. (2020) synthesized a pH-responsive degradable material, acetylated α-cyclodextrin (AaCD), from cyclodextrin α-CD by acetal condensation reaction, which was used as the core of nanocarrier. Then, the cationic material was employed as the shell of nanocarrier, and it composed of PEG chains modified by peptide ligand cRGDfK. And the anti-microRNA-33 (anti-miR33) was loaded on the nanocarrier to obtain pH-responsive nanoparticles (RAAM NPs) (Figure 12A). The anti-miR33 nanotherapeutics significantly promoted reverse cholesterol transport and modulated adaptive immunity by regulating macrophage polarization and T-cell differentiation. Following intravenous administration, RAAM NPs accumulated in AS plaques and associated cells of ApoE-deficient mice via passive and active targeting and released loaded anti-miR-33 molecules in endolysosomes after endocytosis by target cells (Figure 12B), significantly attenuating atherosclerotic vulnerable plaques in mice. Nihad et al. (2022) successfully synthesized pH-sensitive nanoparticles (HR_RAP_NPs) based on hyaluronic acid (HA) by covalently coupling all-trans retinal (ATR) to HA polymers via acid-sensitive hydrazone bonds, and rapamycin (RAP), an anti-atherosclerotic drug loaded into the nanoparticle core, are developed for targeted combination therapy of atherosclerosis. The HR_RAP_ NPs could respond to abnormal pH at the lesion site by breaking hydrazone bond, thereby releasing ATR and RAP at the same time, and simultaneously reduce ROS levels and lipid peroxidation via ATR antioxidant activity and reduce inflammation and inhibit macrophage and SMC proliferation via the anti-inflammatory effect of RAP (Figure 13). More importantly, HR_RAP_ NPs specifically accumulated in atherosclerotic plaques with apolipoprotein E-deficiency (ApoE^−/−^) mice, and remarkably inhibited the progression of atherosclerosis in ApoE^−/−^ mice.

Tang et al. (2015) mixed tannic acid (TA), iron, and polyethylene glycol-based amphiphilic block copolymer (PS-b-PEG) and utilized fast nanoprecipitation (FNP) to generate size-controlled coordination nanoparticles. These nanoparticles were subsequently coated with the tri-ligand octahedral complex TA-Fe^3+^ that exhibited solubility under acidic conditions. Furthermore, the complexes were co-precipitated with hydrophobic fluorescent dyes to produce fluorescent nanoparticles. *In vitro* experiments demonstrated that the nanoparticles had low cytotoxicity and antioxidant activity and promoted intracellular antioxidant delivery.

#### 3.2.2 Intermolecular force-initiated

In addition to covalent bonding, electrostatic interactions, hydrogen bonding, π-electron interactions and supramolecular interactions are also triggering factors for smart drug release from nanocarriers.

The magnitude of these intermolecular forces is modulated by the surface chemical state of the nanomaterial and the pH value of the medium, making them a commonly utilized source for the stimulatory signals of drug nanocarriers (Sánchez-Sánchez et al., 2015). Polyacrylic acid (PAA) is a typical pH-responsive substance, which is prone to protonation under acidic conditions, resulting in a reduction of the electrostatic gravitational force between PAA and certain drug molecules, thereby triggering drug releaseing. Some nanoparticles with pH-responsive charge reversal properties achieve controlled drug release from nanocarriers through altering electrostatic interactions. Drug molecules containing electronegative elements such as fluorine, oxygen, and nitrogen are prone to form hydrogen bonds with the surface-functionalized nanocarriers, and when the pH is changed, the hydrogen bonds are broken due to the change in structure, resulting in drug loading and controlled release. In the actual intermolecular force modulation process, electrostatic interaction and hydrogen bonding sometimes coexist and play a synergistic effect on drug releasing. In addition, intermolecular π-π stacking effects and host-guest interactions also play an active role in the pH stimulation response process. Such pH-responsive nanoagents are widely used in the treatment of tumors (Wang et al., 2022; Cheng et al., 2022; Li et al., 2022; Ma et al., 2022; Rui et al., 2022; Fei et al., 2023), but studies on their application in the diagnosis and imaging of AS are scarce. Nevertheless, the study of drug release mechanisms triggered by intermolecular forces continues to pique the interest of researcher to designing pH-responsive nanocarriers for drugs or genes in AS diagnosis and treatment.

#### 3.2.3 Physical structure change-initiated

Certain polymers have the advantages of adjustable structure and stable in physiological environment, yet they undergo degradation or swelling when exposed to acidic environment, leading to a porous physical architecture. And then, the drugs loaded in the polymers was released, such as chitosan (Obireddy and Lai, 2022). Chitosan is an alkaline hydrophilic polymer that is biocompatible, bioadhesive and biodegradable. Its molecule contains amino and carboxyl groups with pKa of about 6.5 and 2.9, respectively. In a medium with pH 2.5–6.6, chitosan undergoes swelling due to protonation of the amino group in its molecule. Therefore, chitosan and its derivatives are frequently utilized as pH-responsive nanocarriers in various applications. For example, Takechi-Haraya et al. (2015) designed a molecular complex consisting of β-cyclodextrin-grafted chitosan (BCC) and cellular cholesterol efflux-enhancing peptide (CEEP). Compared with β-cyclodextrin, BCC had a significant ability to induce cellular membrane cholesterol efflux, and the BCC-CEEP complex showed twice as much cellular cholesterol efflux as BCC under weakly acidic conditions. Under acidic pH conditions, the high affinity binding of CEEP to BCC resulted in positively charged surface of the 100 nm nanoparticles, which effectively interacted with the cell membrane to induce cholesterol efflux.

### 3.3 Enzyme responses

Many enzymes are involved in the formation process of AS, such as matrix metalloproteinases (MMPs), hyaluronidases, and cathepsins, which also could be used as the stimulators and targets for drug delivery and controlled release in atherosclerosis treatment. At present, MMPs and hyaluronidase are the most extensively researched stimulators and targets, of which MMP13, MMP2, and MMP9 are three common target metallo-mechanism proteases. Additionally, cathepsin B has significantly higher activity in unstable plaques than in stable plaques and may be used as both a target and a stimulator of AS (Tawakol et al., 2016).

#### 3.3.1 Matrix metalloproteases response

Matrix metalloproteinases (MMPs) are produced by a variety of cells including pro-inflammatory cells, fibroblasts, endothelial cells, VSMCs and macrophages, and play an important role in maintaining normal vascular architecture. In contrast, the formation of AS is influenced by MMPs which modulate endothelial cells function, promote migration and proliferation of VSMCs, and contribute to angiogenesis, apoptosis, and tissue repair (Opincariu et al., 2021). In addition, MMPs are typical protein hydrolases that degrade various protein substrates (e.g., collagen, elastin, and fibronectin) in the ECM, leading to instability and rupture of AS plaques (Mina and Raouf, 2012; Olejarz et al., 2020). Therefore, MMPs serve as both stimulators and targets for AS treatment. For example (Qin et al., 2016), combined gold nanorods with MMP2 antibodies to obtain a highly efficient photoacoustic imaging (PAI) probe (AuNRs-Abs) and scanning electron microscopy and immunofluorescence showed that AuNRs-Abs targeted specifically MMP2, which was used for localization of AS plaques and quantification of MMP2. In addition, Nazanin et al. (2017) developed several MMP2/MMP9 inhibitors labeled with ^123^I that were used for single photon emission computed tomography (SPECT) imaging, while effectively targeting mouse AS lesions with high selectivity and inhibitory potency.

#### 3.3.2 Hyaluronidase response

**FIGURE 14:**
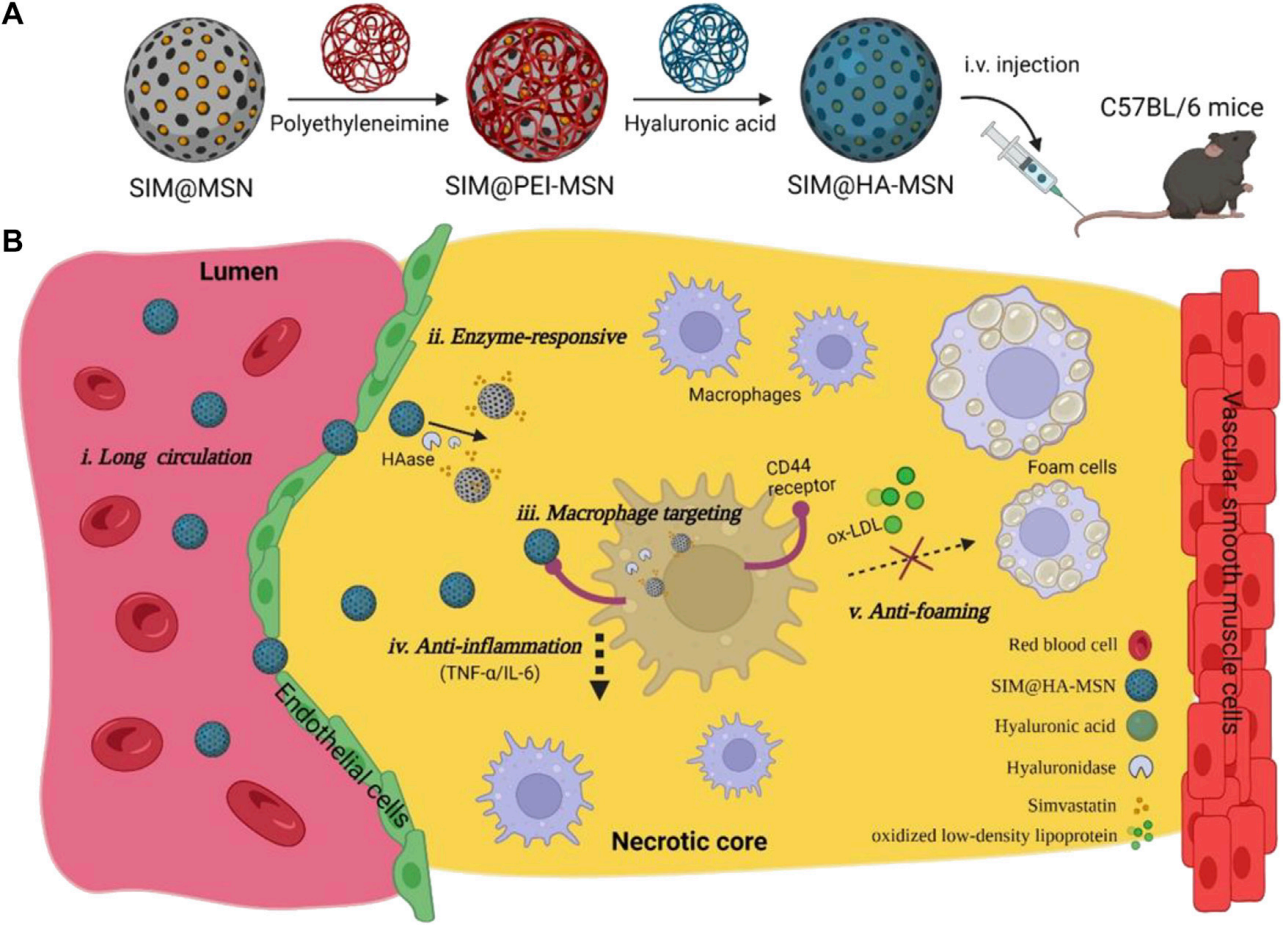
Schematic illustration of construction of SIM@HA-MSN for atherosclerosis management. **(A)** Scheme of fabrication process and administration route of SIM@HA-MSN. **(B)** Illustration of SIM@HA-MSN therapy which can potentially alleviate atherosclerosis through long circulating, enzyme-responsive drug release, macrophage targeting, and anti-inflammatory and anti-foaming effects (Song et al., 2022).

The results of study indicated a significantly elevated concentration of hyaluronidase (HAase) in AS plaque compared to normal tissue, which contributed to the instability of AS plaque. HAase is an enzyme that specifically degrades hyaluronic acid (HA), while HA and the proteins bound to it regulate the inflammatory process and tissue injury and repair by regulating the recruitment of inflammatory cells, the release of inflammatory factors and the migration of stem cells (Kim et al., 2019). Moreover, HA is a non-collagenous component of the ECM and plays a crucial role in tissue injury response (Jiang et al., 2007; Wight, 2016), HA also recognizes and binds to CD44 receptors expressed on inflammatory macrophages. Therefore, coating the drug with HA could achieve dual effects: targeted binding to CD44 receptors and degradation by HAase at AS lesions, leading to drugs release and further inhibition the development of AS. For example, Zhang et al. (2017) designed an HA-anchored core-shell structured nanoparticle, which consisted of a PLGA core and a surface-coated lipid layer containing HA-anchored rHDL. The nanoparticles were effectively stabilized in the blood environment. While the HA coating on the surface of the coating reduced hepatic uptake and targeted the CD44 receptor, which was over-expressed by leaky endothelial cells. Subsequently, macrophages took up the nanoparticles and release rHDL in the presence of hyaluronidase to remove cholesterol. *In vivo* studies have shown that such r-HDL delivery nanoparticles reduced total cholesterol, low-density lipoprotein cholesterol (LDL-C) and triglyceride levels, increased high-density lipoprotein cholesterol (HDL-C) levels, and reduced plaque area. Song et al. (2022) constructed a nanoparticle (SIM@HA-MSN) with enzyme-responsive and macrophage-targeting properties. The lipid-lowering drug simvastatin (SIM) was loaded into mesoporous silica nanoparticles (MSN), which were then coated with HA to confer HAase responsiveness and inflammatory macrophage targeting (Figure 14). The results showed that MSN nanocarriers exhibit high loading efficiency (>20%) and excellent enzyme responsiveness. In addition, *in vitro* experiments confirmed the targeting and anti-inflammatory effects of SIM@HA-MSN with low cytotoxicity and good hemocompatibility. Animal studies revealed prolonged plasma retention time and favorable *in vivo* biocompatibility of SIM@HA-MSN.

#### 3.3.3 Cathepsins response

**FIGURE 15:**
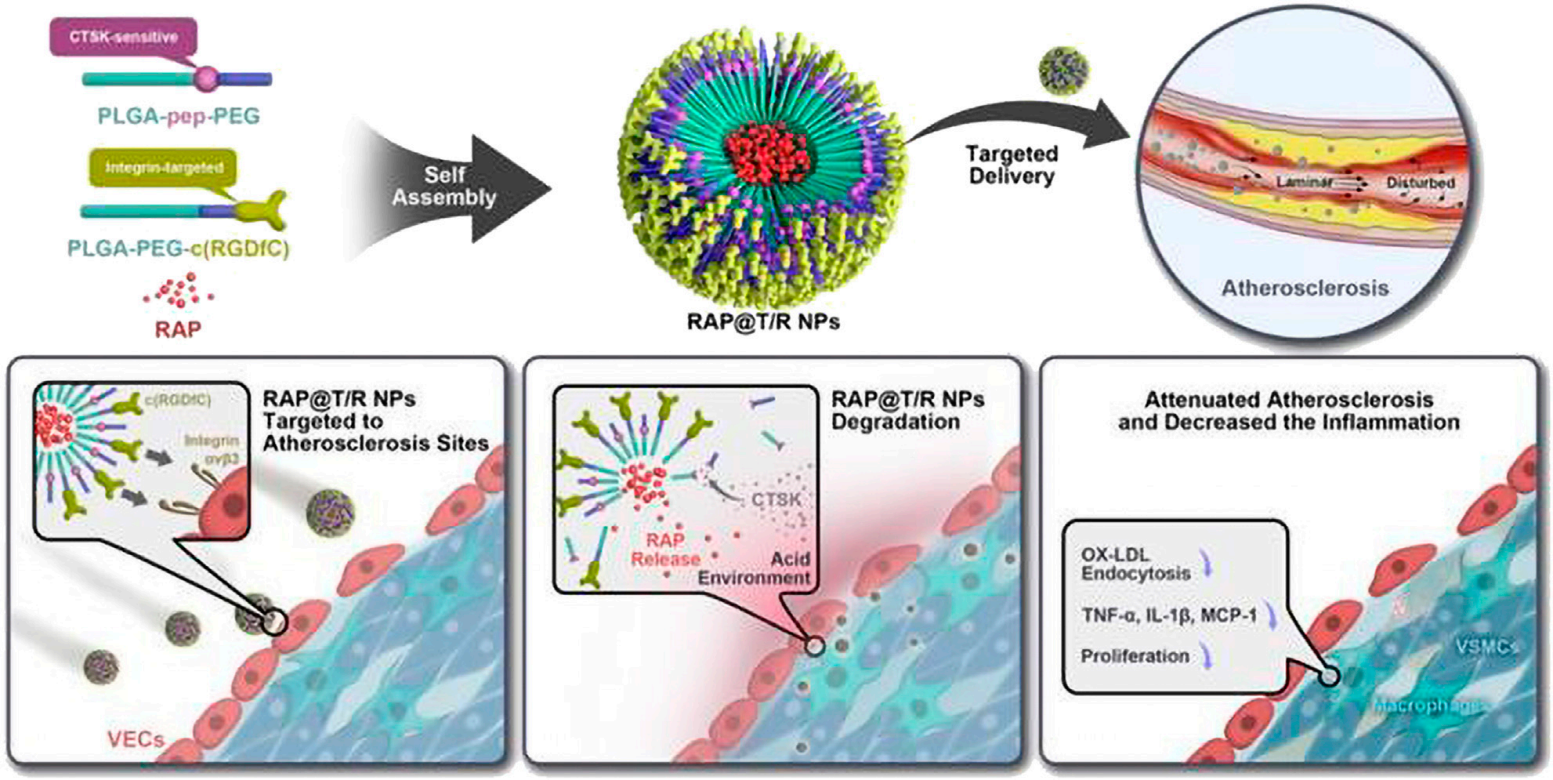
Schematic diagram of the components of the RAP@T/R NPs and targeted delivery RAP to treat atherosclerosis in response to CTSK (Fei et al., 2022).

Cathepsins are a class of protein hydrolases that are expressed in eukaryotic cells and primarily located within lysosomes. Studies have demonstrated that cathepsins are involved in the formation of AS, in physiological processes such as infiltrative migration of macrophages, migration of VSMCs, apoptosis, and inflammatory responses (Shintaro et al., 2018; Jia et al., 2019; Zhang et al., 2019). Moreover, the activity of cathepsin B is elevated in unstable plaques compared to stable ones (Ihab et al., 2016). Therefore, cathepsins serve as potential targets for drug therapy or stimulation points for drug delivery. Shon et al. (2013) found that after intravenous injection of cathepsin B-activating therapeutic agent (L-SR15) in AS mice, L-SR15 was cleaved and released fluorescent agent (chlorine-e6). The released chlorine-e6 was used to selectively eliminate macrophages and attenuate cathepsin B activity in unstable plaques, thus enabling diagnostic visualization and therapeutic applications. Based on the findings of enrichment of cathepsins k (CTSK) enzymes in atherosclerotic lesions, Fei et al. (2022) designed CTSK-sensitive and integrin αvβ3-targeted nanoparticles (RAP@T/R NPs) to deliver RAP for the treatment of atherosclerosis. The studies showed that RAP@T/R NPs possess the ability to selectively target atherosclerotic lesions by binding to αvβ3 overexpressed in inflammatory VEC. *In vitro* experiments revealed that CTSK stimulation could accelerate the release of RAP from NPs, which significantly inhibited phagocytosis of OxLDL and cytokine release from inflammatory macrophages. Furthermore, T/R NPs prolonged blood retention and increased accumulation in atherosclerotic lesions. RAP@T/R NPs significantly impeded the development of atherosclerosis and suppressed both systemic and local inflammation in ApoE^−/−^ mice. Figure 15

### 3.4 Shear response

Vessels are exposed to a diverse array of hemodynamic forces, including fluid shear stress, hydrostatic pressure, pulsatile blood pressure and tensile stresses induced by circulating blood flow (Chiu and Chien, 2011). As the AS plaque continues to develop, luminal narrowing occurs and there is an increase in fluid shear and wall shear stress (WSS) at the site of the lesion. The normal range of WSS in vessels is 1–10 dyn cm^-2^, whereas in vulnerable plaque vessels, it increases to a range between 31.90 and 136.09 dyn cm^-2^ with significantly higher values observed at the proximal end (approximately 180 dyn cm^-2^) compared to the distal end (approximately 100 dyn cm^-2^) (Korin, 2012; Chen et al., 2017). This abnormal shear stress could act as a stimulating and targeting factor, triggering local drug delivery and released to the target lesion plaque lesion. Currently, the majority of micro-nanoparticles utilized for drug delivery via fluid shear stimulation are designed based on platelets structure mimicry, with their drug release primarily attributed to carrier deformation or degradation under high shear conditions. For example, Margaret et al. (2012) developed liposomal nanoparticles with a lentilshaped that can undergo deformation under high shear stress, facilitating the release of loaded drugs. Korin (2012) prepared a microscale aggregation of nanoparticles (SA-NTS) that can be activated by shear stress, mimicking the structure of platelets, and loaded the thrombolytic drug tissue-type fibrinogen activator (tPA) into SA-NTS. The SA-NTS released drug to the obstructed vessel using the high shear stress caused by vascular stenosis. *In vitro* experiments revealed that under abnormally high fluid shear stress, SA-NTS disintegrated into individual nanofractions. When SA-NTS loaded with tPA were injected intravenously in mice, these shear-activated nanotherapeutic drugs induced rapid clot lysis in a model of mesenteric injury, restored normal hemodynamics, and increased survival in pulmonary embolism mouse model.

### 3.5 Exogenous stimuli-responsive and multi-stimuli-responsive

Currently, researchers have developed a range of exogenous stimuli-responsive nanoagents, primarily composed of inorganic nanoparticles commonly used for *in vitro* diagnosis of AS diseases. These agents are activated by various exogenous stimuli such as light, ultrasoundand magnetic fields. These nanoagentsare activated in response to specific exogenous stimuli for imaging and detected by corresponding devices. Some inorganic nanoagentsare capable of imaging along with the diagnostic integration, such as gold nanoparticles (Kharlamov et al., 2015), ions nanoparticles (Wang et al., 2013), and copper sulfide (Gao et al., 2018) nanoparticles. In addition, the integration of these exogenous stimuli-responsive inorganic nanoparticles with organic counterparts can enable drug delivery while simultaneously fulfilling the diagnostic imaging function of inorganic nanoparticles, thereby achieving multiple potential functionalities.

Multi-stimuli-responsive nanoagents utilize multiple endogenous or exogenous stimuli to target the lesion site for intelligent therapy, and the multi-stimulus response enhances the performance of nanoagents with superior targeting and higher drug delivery efficiency. There is a limited number of studies on multi-responsive nanotherapeutic agents targeting AS, and the majority of multi-responsive nanoplatforms are constructed based on light response, such as light/ROS response (Jager et al., 2016), light/pH response (Hong et al., 2018), and light/enzyme response (Shon et al., 2013). Other nanoagents that respond to multiple stimuli, such as those combining pH, shear stress, and ROS (Dou et al., 2017; Peters et al., 2019), hold great potential for treating AS.

## 4 Conclusion and perspectives

The theranostic nanomedicine employs nanotechnology to design and fabricate targeted drug (gene) delivery or multi-responsive functional nanocarriers, as well as to develop novel nanoagents and drugs, allows us to fight against diseases with complex pathologies such as AS and tumors at the molecular level. Based on the pathological characteristics of AS, the construction of nanoagents with delayed drug release, prolonged *in vivo* circulation and targeted drug delivery is the key to achieve efficient and low-toxicity treatment of AS. By modifying their surface, nanoparticles are able to evade the recognition and clearance of the immune system *in vivo*, target the over-expressed receptors in the lesion and bind to them to inhibit the development of the disease, or under the stimulation of abnormal microenvironment (ROS, enzymes, pH and shear stress) in the lesion, the nanocarriers disintegrate and release anti-inflammatory drugs or disrupt the abnormal gene sequences. Therefore, nanoagents hold immense potential in the treatment of AS, making theranostic nanomedicine a promising avenue for its management. However, to enhance the delivery efficiency and safety of these agents, it is imperative to delve deeper into the pathogenesis and pathology of AS while also identifying viable drug delivery targets. In addition, the synthesis methods and drug release mechanisms of different responsive nanoagents for the abnormal microenvironment of AS lesion sites need to be further improved and refined.
